# An Update on Retinal Diseases: From Diagnosis to Treatment

**DOI:** 10.3390/jcm15093273

**Published:** 2026-04-24

**Authors:** Shigeru Honda, Masahiko Sugimoto, Kenji Yamashiro

**Affiliations:** 1 Department of Ophthalmology and Visual Sciences, Osaka Metropolitan University Graduate School of Medicine, Osaka 545-8585, Japan; 2 Department of Ophthalmology and Visual Science, Faculty of Medicine, Yamagata University, Yamagata 990-8585, Japan; 3 Department of Ophthalmology and Visual Science, Kochi Medical School, Kochi University, Nankoku 783-8505, Japan

Retinal disease is one of the major causes of acquired blindness in most developed countries [1]. In particular, cases of retinal disease are increasing amongst elderly individuals, and they need appropriate treatments to maintain good vision for better quality of life. Recent advancements in medical technology have enabled us to disclose the etiologies of several common and rare retinal diseases, and some new diagnostic criteria and treatment modalities have been developed [2,3]. Investigations into the mechanisms of retinal diseases and the development of new modalities to treat them are progressing day by day. In the meantime, gathering and reviewing the latest knowledge is also mandatory to acquire a better understanding of such diseases. Here, we release a Special Issue: “An Update on Retinal Diseases: From Diagnosis to Treatment”. The purpose of this issue is to invite original articles and review papers regarding the diagnosis of and intervention in retinal disorders, which will serve the readers in this field to better manage patients with those pathologies.

We sincerely appreciate all the authors who published their outstanding works in this Special Issue, comprising 16 manuscripts (14 original articles, one review paper, and one brief report), providing the latest unique knowledge regarding the diagnosis and treatment of retinal diseases, including neovascular age-related macular degeneration (nAMD) (five articles), diabetic macular edema (DME) (three articles), retinal vascular occlusion (two articles), central serous chorioretinopathy (two articles), retinitis pigmentosa (one article), and retinal degenerative side effects of drugs used for systemic diseases (two articles).

To mention a specific topic, anti-VEGF therapy is the first-line treatment for many kinds of exudative retinal diseases [4], and recently, second-generation anti-VEGF agents (brolucizumab, faricimab, aflibercept 8 mg) have been developed. However, most phase 2/3 clinical trials have included only treatment-naïve patients, failing to reveal the effects of such anti-VEGF agents in a wider range of patients, including pre-treated or refractive cases. Hence, accumulation of real-world data is vital to evaluate the true efficacy of new treatment modalities. In this Special Issue, some studies reported the outcomes of the second-generation anti-VEGF agents brolucizumab and faricimab in nAMD or DME in real-world clinical settings [5,6,7,8]. These papers also highlight new diagnostic findings and factors that affect the treatment outcomes in these disorders. There exist multiple kinds of anti-VEGF agents with their own unique characteristics (e.g., molecular size, concentration, affinity, add-on effect, prices) in addition to an anti-VEGF effect that enables physicians to choose the most appropriate option for each patient. In the meantime, every anti-VEGF agent may also have some considerable disadvantage, and some of these (e.g., intraocular inflammation (IOI)) have already been shared among many ophthalmologists. However, other unknown risk factors should be considered in selecting the appropriate anti-VEGF agent for certain patients. Yamamoto M et al. evaluated the immediate change in intraocular pressure after intravitreal injections of five anti-VEGF agents and reported different outcomes among the agents tested, which should be considered when delivering anti-VEGF therapy to patients who are sensitive to IOP elevation [9]. Recent progress in artificial intelligence (AI) technologies is outstanding, revealing many aspects of retinal diseases that were not recognized by traditional ocular examinations, or proposing a better monitoring procedure for disease activity in several retinal disorders [10]. Currently, macular fluid is recognized as a negative sign for better vision in diabetes patients, but it is difficult to conduct a quantitative analysis to predict patients’ vision. Mitamura H et al. presented AI-based macular fluid parameters associated with visual acuity in diabetic macular edema (DME), which may be used as an automated disease-activity measuring modality in future clinical settings to monitor and treat DME [11].

Central serous chorioretinopathy (CSC) is a representative pachychoroid disease that presents with serous subretinal fluid at the macula with no detectable macular neovascularization (MNV) [12,13]. It is widely known that CSC phenotypes have wide ethnic and individual variations, which suggests its complex pathophysiology. CSC is associated with certain choroidal vascular abnormalities (flow voids in the choriocapillaris accompanied by dilated Haller-layer vessels and vortex veins), but the detailed mechanisms of these phenomena have not been clarified yet. Sugiyama R et al. evaluated the change in choroidal circulation time before and after half-dose photodynamic therapy in CSC [14]. Their findings are interesting in terms of understanding the regulation mechanism of choroidal blood flow, which may be involved in the pathophysiology of this complex disease. As a treatment for CSC, sub-threshold laser therapy is often used, but the identification of irradiated spots is a major problem of this modality [15]. Jeon SH et al. reported a fundus image-based titration strategy for Selective Retina Therapy for CSC [16].

In retinal vascular diseases, retinal artery occlusion is one of the most emergent ophthalmic conditions, and often causes irreversible visual impairment [17]. Many challenges have been reported in treating this disorder, but no robust consensus has been established to date. Roskal-Wałek J et al. published a review article regarding therapeutic strategies for retinal artery occlusion (https://doi.org/10.3390/jcm13226813), which is very helpful in understanding and maintaining this emergency condition [18]. In addition, a report in this Special Issue introduced gradient-weighted class activation mapping in fluorescein angiography images to predict long-term visual outcomes in branch retinal vascular occlusion.

Regarding the retinal degenerative side effects of drugs used for systemic diseases, this Special Issue reports on subclinical detection of hydroxychloroquine-induced retinopathy using multifocal electroretinography and optical coherence tomography (https://doi.org/10.3390/jcm13247663), as well as real-world practices of pentosan polysulfate maculopathy (https://doi.org/10.3390/jcm13175090). These reports provide new insights into this research area and give physicians an important point of observation in clinical practice.

In summary, this Special Issue contains many new insights into several retinal diseases. We hope this Special Issue will be useful to readers seeking a better understanding of the management of patients with retinal diseases.
